# STEP-HI Study: A Multimodal Intervention of Exercise and Testosterone Therapy: Design and Real-World Challenges

**DOI:** 10.1093/geroni/igab046.1142

**Published:** 2021-12-17

**Authors:** Ellen Binder, Jay Magaziner

## Abstract

Hip fractures are common among older women and can have a devastating impact on their ability to remain independent. Many women who were high functioning before the hip fracture do not return to their pre-fracture level of function, have persistent weakness and mobility impairments, and may require ongoing supportive services. Age-associated androgen deficiency may contribute to deficits in muscle mass, strength and power that are common in older female hip fracture patients. The Starting a Testosterone and Exercise Program after Hip Injury (STEP-HI ) Study is a three-group, randomized, double-blinded, placebo-controlled Phase III clinical trial designed to evaluate a multi-modal intervention aimed at improving functional outcomes in older female hip fracture patients. 168 female hip fracture patients, age 65 yrs. and older are being recruited from 6 clinical sites in the USA. Participants are being assigned to one of three groups: supervised exercise (EX) plus 1% testosterone topical gel; EX plus placebo gel; or enhanced usual care. The primary outcome is six-minute walk distance. This symposium will present information related to key aspects of the design of the STEP-HI trial, including recruitment of a frail patient population during a period of injury recovery, the testosterone and exercise interventions and related fidelity procedures, and implementation challenges for this multi-modal intervention prior to and during the COVID-19 pandemic. In addition, the underlying mechanisms by which testosterone and exercise are expected to have a synergistic effect on muscle strength and function will be discussed.

